# Assessing Affective Valence and Activation in Stretching Activities with the Feeling Scale and the Felt Arousal Scale: A Systematic Review

**DOI:** 10.1177/00315125231160203

**Published:** 2023-03-01

**Authors:** Leonor Henriques, Diogo S. Teixeira

**Affiliations:** 1 Faculty of Physical Education and Sport, 70887 Lusófona University*,* Lisbon, Portugal; 2 Research Center in Sport, Physical Education, and Exercise and Health (CIDEFES)*,* Lisbon, Portugal

**Keywords:** core affect, feeling, arousal, exercise, measurement

## Abstract

Affective responses have been considered key determinants for exercise adherence, but research on affective responses to stretching activities is scarce. Given the role of these responses in exercise adherence, our aim in this review was to explore (a) the utility and feasibility of core affect in stretching-related activities as measured by the Feeling Scale (FS) and/or the Felt Arousal Scale (FAS); (b) the timing of administering these scales; and (c) the scales’ applicability and interpretability in this context. Inclusion criteria for studies in this review were experimental and non-experimental studies written in English that based affect assessment on the FS and/or FAS and that applied these scales to participants engaged in physical activity, individually or in groups. We also considered studies that focused on stretching activities that were either isolated or components of a class/activity and studies that used healthy participants of any age. Exclusion criteria were populations with mental health problems, cancer, diabetes, hypertension, cardiovascular disease, or diseases likely to alter pain perception or be associated with chronic pain, instrument validation studies, gray literature, and systematic reviews. We searched PubMed, SPORTDiscus and PsycINFO databases, and we added studies retrieved manually from reference sections while following PRISMA guidelines. We used the Effective Public Health Practice Project tool for judging methodological quality of research articles. Our final analyses were based on 12 empirical studies published between 2003 and 2021with a total of 718 participants. Both scales were found to be useful and feasible in the most usual places for exercise, but core affect results cannot be properly interpreted due to variability of study protocols and the absence of guidelines for adequate baseline assessment. Most studies recorded affect responses pre-session, during session, and post-session. We observed no standardized timing or frequency of assessment, and there was high heterogeneity among stretching protocols. Currently, research in core affect assessment of stretching-related activities lacks sufficient methodological quality to draw generalizable conclusions.

## Introduction

There is now irrefutable evidence of the effectiveness of regular physical activity (PA) in the primary and secondary prevention of several chronic diseases (e.g., cardiovascular disease, diabetes, cancer, hypertension, obesity, osteoporosis, depression, and anxiety) and premature death (Warburton & Bredin, 2017; Warburton et al., 2006). Still, 36.2% of adults in Europe aged 18 and over were not sufficiently active in 2017 (Nikitara et al., 2021) in that they did not meet the World Health Organization recommendations of at least 150 minutes of moderate-intensity, or 75 minutes vigorous-intensity physical activity per week. If current trends continue, the 2025 global physical activity target (a 10% relative reduction in insufficient physical activity) will not be met (Guthold et al., 2018). These circumstances reveal the importance of understanding how to successfully increase PA levels among the general adult population.

Several psychological frameworks in exercise psychology have been put forth to explain and increase sustainable PA behavior, including social cognitive theory (Bandura, 1998), the theory of planned behavior (Ajzen, 1991), and the transtheoretical model of behavior change (Prochaska & Velicer, 1997), just to name a few. However, these theories have been criticized for their heavy emphasis on cognitive and reasoned bases of behavior that overlook or reject the contribution of non-rational processes in decision making (Ekkekakis & Zenko, 2016; Rebar et al., 2016). Thus, during the last decade, affect, an umbrella term for general valenced experiential responses (i.e., core/basic affect), emotions, and mood (Ekkekakis & Petruzzello, 2000), has been increasingly represented as a central aspect of behavior maintenance/change that is grounded on hedonic assumptions. Affect has been explored either as a reflective process (e.g., affective judgments, like enjoyment) or an automatic process (e.g., affective association) in several dual-process theoretical approaches related to exercise and/or health-related behavior. These include the Affective-Reflective Theory (ART; Brand & Ekkekakis, 2018), the Physical Activity Adoption and Maintenance model (PAMM; Strobach et al., 2020), the Affective and Health Behavior Framework (AHBF; Williams & Evans, 2014), and the Theory of Effort Minimization in Physical Activity (TEMPA; Cheval & Boisgontier, 2021), as examples. Particularly in the AHBF, the immediate affective response (e.g., during running) and the post-behavior affective response (e.g., immediately after a workout) are considered key determinants of healthy behavior. This affective response can be understood as core affect (i.e., an elementary non-reflective feeling consciously available), and it has demonstrated relevant predictive value for exercise adherence (Rhodes & Kates, 2015; Rodrigues et al., 2020; Williams, 2008). However, exercise prescription guidelines emitted by international entities have failed to describe how to assess individual affective states, judgments, or associations, or how to adjust exercises or exercise sessions to promote a better affective response (e.g., American College of Sports Medicine).

Basic affective responses to exercise have been commonly, easily, and reliably assessed by the Feeling Scale (FS; Hardy & Rejeski, 1989) and the Felt Arousal Scale (FAS; Svebak & Murgatroyd, 1985), depicting the participant’s affective valence (perceived pleasure/displeasure) and arousal (perceived activation), respectively (Evmenenko & Teixeira, 2020). Importantly, affective responses can be plotted into the circumplex model of affect (Ekkekakis et al., 2011; Russell, 1980), which allows tracing the responses in different exercise modalities or activities, thus permitting the assessment of affective responses to emerge as a powerful means for exercise professionals to refine their prescriptions. To date, affective responses to exercise have been mainly studied in aerobic and resistance exercises (Cavarretta et al., 2019; Evmenenko & Teixeira, 2020), with only scarce attempts to apply them to stretching activities (e.g., yoga, tai-chi, Pilates) that have recently gained worldwide popularity (Thompson, 2022; Yeh et al., 2008, 2009). This is important, not only because stretching activities have long been used within many physical exercise activities to increase range of motion around a joint (Opplert & Babault, 2018; Riebe et al., 2018), but also because they are usually employed and integrated in exercise warm-up or recovery stages, or even as the main component of several group fitness classes. Stretching exercises are directly related to improvements in self-rated sleep quality (Smith et al., 2007; Telles et al., 2012; Yoshihara et al., 2014), flexibility and dynamic balance, muscular endurance (Cruz-Ferreira et al., 2011), and cardiorespiratory fitness (Fernández-Rodríguez et al., 2019), and they have been associated with or led to improvements in self-esteem, health-related quality of life, and mood (Smith et al., 2007; Telles et al., 2012; Yoshihara et al., 2014) and to reductions in perceived stress, anxiety, depression (Wang et al., 2010), and pain (Miyamoto et al., 2013). Stretching activities are considered to be secure, accessible, and engaging (DiGiacomo et al., 2010), and they are likely contributors to good physical activity adherence rates (Chao et al., 2014; Yeh et al., 2009).

In this context, understanding how to promote perceived pleasurable experiences from engagement in stretching activities may allow participants a better adjustment to these activities and their desired outcomes by supporting exercise engagement and adherence. More specifically, analyzing known challenges to the specific use of the FS and FAS for assessing affect might include attention to (a) the utility and feasibility of core affect assessment with these tools when applied to stretching-related activities, (b) the timing of assessments with these tools, and (c) the contextual applicability and interpretability of these measures. We focused this systematic literature review on these themes.

## Method

We prepared this review by following recommendations suggested by the Preferred Reporting Items for Systematic Reviews and Meta-Analyses (PRISMA) protocol (Page et al., 2021), and registered our review in the international Prospective Register of Systematic Reviews (PROSPERO) with the following number: CRD42022329331. We were also guided by the Population, Intervention, Comparison, Outcomes and Study (PICOS) strategy. Both the PRISMA guidelines and the PICOS tool are endorsed by the Cochrane Collaboration (Higgins & Green, 2011; Methley et al., 2014), and PROSPERO registration has been widely endorsed for transparency purposes (Booth et al., 2012). The means by which we adhered to these varied guidelines is detailed below.

### Eligibility Criteria

We adopted the following inclusion criteria for studies in this review: (a) experimental and non-experimental studies; (b) studies written in English; (c) studies that based affect assessment on the FS and FAS and applied these scales to both individual and group physical activity settings; (d) studies that strictly used stretching activities that were either isolated or components of a class/activity; (e) studies with participant samples of any age (young adults, adults, older adults); and (f) studies focusing on apparently healthy individuals. Exclusion criteria were as follows: (a) study populations with mental health issues; (b) study populations with cancer, diabetes, hypertension, cardiovascular disease, or diseases likely to alter the perception of pain or with chronic pain; (c) instrument validation studies; (d) gray literature (e.g., thesis, conference abstracts, reports); and (e) systematic reviews. Level I study screening included a review of all titles and abstracts to check those against eligibility criteria. Full-text publications of any studies not eliminated at Level I were retrieved for complete review at Level II screening; this involved reading the full-text publication to determine that all eligibility criteria were met, and no exclusion criteria were applicable.

### Information Sources and Search Strategy

A wide search of scientific papers was conducted from January 2022 until February 2022, on the following databases: PubMed (host: MEDLINE; last search run February 2022), SportDISCUS (host: EBSCO; last search run February 2022) and PsycINFO (host: EBSCO; last search run February 2022). The search used the following entries: “physical activity”, “physical exercise,” “Feeling Scale,” “Felt Arousal Scale,” “flexibility,” and “stretching.” These keywords were searched separately and in various combinations with the use of conjunctions such as “AND” and “OR,” organized according to PICOS strategy. Bibliographic references from related studies and other sources were also examined to include studies that potentially met the inclusion criteria (last search conducted February 2022).

### Data Collection and Data Items

For a general characterization of these retrieved studies (Table 1), we extracted the following main data characteristics: (a) bibliographic information (authors, year of publication, country of research), (b) study design, (c) sample size, (d) sample characteristics, (e) FS/FAS measures, (f) analysis, and (g) outcomes.

**Table 1.** General Descriptive Characteristics of the Studies Reviewed and Their Main Outcomes.

Author(s)	Location	Design	Size (%F)	Features (Age M ± SD)	Intervention	Measures	Analysis	Outcomes	Quality
Barbosa et al. (2018)	Brazil	Randomized controlled trial	*n* = 45 (0)	Control group (CG): (21.27 ± 2.8). Static stretching group (SSG): (23.07 ± 3.5). Dynamic stretching group (DSG): (21.47 ± 3.0)	CG: no intervention. SSG: 3 × 30″ sets of hamstring self-stretching for each limb, mild discomfort; DSG: Dynamic hip flexion with knee extension 3× 30″ sets for each limb.	FS post-exercise	Chi-squared test	Chi-squared test showed no statistically significant association between experimental groups and sensation during stretching.	Moderate
Beltrão et al. (2020)	Brazil	Randomized controlled trial	*n* = 14 (0)	Untrained men (21.53 ± 2.52)	Hamstrings stretch: 3 × 60″ with 30″ rest intervals. 3×/week, for 12 weeks. Low-intensity group (LIG): Intensity between 1 and 2 of verbal numerical scale. High- intensity group (HIG): Intensity between 9 and 10 of verbal numerical scale.	FS pre-intervention, 6 weeks, and 12 weeks.	Mann–Whitney U test Friedman test	The Mann–Whitney U test for affective responses showed no significant differences between the groups at preintervention, intermediate and final assessments. The Friedman test showed no differences in affective responses between moments for both LIG and HIG.	Strong
Chao et al. (2014)	Brazil	Quasi-experimental	*n* = 16 (100)	Experienced practitioners (61.2 ± 8.8)	Tai-Chi 3×/week for 7 weeks.	FS at 15′, 30′, and 45′ assessed in each session (21 sessions).	Friedman test with Dunn's post *hoc* test.	There was a significant difference between responses at 15′ and 30’; and between responses at 15′ and 45’. There was no difference in responses between 30′ and 45′ min.	Weak
Edwards et al. (2018)	United States & Canada	Randomized control experimental	*n* = 63 (0)	Walking group (WG) (*n* = 21): (21.24 ± 2.17); meditation group (MG) (*n* = 21): (21.29 ± 2.10); stretching group (SG) (*n* = 21): (21.52 ± 1.75)	SG: 10′ of guided stretching. 10 upper and lower extremity stretches, each stretch was performed twice, for 20–30”; WG: 10′ brisk walking; MG: 10′ guided mind- fulness meditation.	FS & FAS pre, during, post-exercise	3 × 2 ANOVAs	SG and WG increased FS scores significantly. WG increased arousal significantly.	Weak
Follador et al. (2019)	Brazil	Quasi-experimental	*n* = 70 (100)	Experienced practitioners. Tai-chi group (TCG) (68.65 ± 8.04); Yoga group (YG) (70.04 ± 6.65); stretching group (SG) 69.47 ± 5.50)	60 min classes 2×/week. 5′ of warm-up exercises; 50′ of specific techniques (Tai-chi, yoga, or stretching); and 5′ cooldown exercise.	FS & FAS at baseline, 15′, 30′, 45′, 60’	ANOVA; Shapiro–Wilk test; Kruskal–Wallis test, followed by the Mann–Whitney post hoc test with a Bonferroni correction. Cohen’s d The effect of time (pre-exercise, 15′, 30′, 45′, 60′) was analyzed with the Friedman test followed by the Wilcoxon post hoc test with a Bonferroni correction.	Affective responses were similar between groups. However, tai-chi results for mean arousal were significantly different compared to the yoga and stretching groups.	Weak
Lee et al. (2021)	South Korea	Quasi-experimental	*n* = 16 (75)	University students (21.2 ± 2.4)	2 Zumba sessions (low and moderate intensity; 45′): 10′ warm-up, 30′ Zumba program, 5′ cooldown.	FS & FAS (pre-exercise, warm-up, and cooldown) using tailor-made applications on a smartwatch.	Two-way repeated measures ANOVA Pearson correlation coefficient between HR and feeling, and between HR and arousal for each exercise section.	FS revealed a significant interaction effect of exercise intensity and section. Post hoc tests indicated that in the low- intensity condition, pre-exercise FS scores were significantly lower than the FS scores measured during exercise and cooldown. However, no differences in FS were observed among the exercise sections. In the moderate-intensity condition, the FS scores of all the exercise sections were significantly higher than that of pre-exercise. The FS scores of reggaeton, cumbia, and cooldown were higher than those of the pre-exercise and warm-up, with no differences across sections. FAS revealed a significant interaction effect of exercise intensity and section. In the comparison of the FAS in the low- intensity condition, the pre-exercise FAS was significantly lower than the FAS during all sections except warm-up, with no differences across sections. In the moderate-intensity condition, the FAS scores of all exercise sections were higher. Compared to pre-exercise. The FAS of warm-up was lower than those of salsa, cumbia, and cooldown.	Weak
McAuley, Jerome, Marquez, et al. (2003)	USA	Randomized controlled trial	*n* = 174 (72)	Older, unfit, and overweight (65.5 ± 5.35)	6-month (3×week) of either: Walking classes (WG) (*n* = 85) (50–55% VO2peak) gradually increasing to (65% VO2peak); stretching & toning classes (STG) for 60’ (*n* = 89) (intensity undetermined)	FS following each exercise session (daily). FS post-exercise responses were averaged over the final (6th) month	Latent growth curve analyses.	The average of the FS responses was higher in the STG than in the WG. Sized effect of exercise frequency on exercise affect was nonsignificant,	Moderate
McAuley, Jerome, Elavsky, et al. (2003)	USA	Longitudinal	*n* = 174 (71,8)	(65.5 ± 5.35)	6-month exercise program + 18 months follow-up	FS following each exercise session (daily). FS post-exercise responses were averaged over the final (6th) month	A 2 (condition) by 2 (sex) repeated measures ANOVA.	Greater social support within the exercise setting was associated with more positive affective responses during the exercise program. The relationship between social support and affect was found to be significant.	Moderate
Melo et al. (2021)	Brazil	Randomized controlled trial	*n* = 41 (0)	No stretching (NS) (23.8 ± 4.1); Comfort level stretching (CLS) (24.7 ± 4.8); Mild discomfort level stretching (MDLS) (24.7 ± 4.8); pain level stretching (PLS) (22.8 ± 2.1)	NS (*n* = 10); CLS (*n* = 11); MDLS (*n* = 10); PLS *n* = 10). 10 sessions of 3 × 30″ sets of passive static stretching 3×/week.	FS post-exercise (48 h after the 10th session)	One-way ANOVA was carried out to compare intergroup sensation of affective valence	Affective valence showed similar and higher positive responses for the PLS and MDLS compared to the CLS group after the static stretching protocol.	Strong
Reed (2014)	Chicago, Illinois, USA	Quasi-experimental	*n* = 45 Experimental group was 70% female and 30% male, and the control group was 50% male and 50% female	Experimental group: (28.55 ± 10.91); Control group: (28.27 ± 7.77)	Control group (CG) 50′ yoga 2× week for 15 weeks. Experimental (placebo) (EG): 50′ yoga 2× week for 15 weeks, with bracelet during class for 6 weeks (weeks 2–7)	FAS baseline (during pre-relaxation in week 1 & 2), acute placebo (Affect was measured during final relaxation in week 2), 6-week placebo (week 7), acute no-placebo (week 8), and 6- week no-placebo (week 13).	2 × 5 (group × placebo phase) repeated measures ANOVA	Neither group reported significant FAS changes.	Weak
Sullivan et al. (2019)	USA	Quasi-experimental	*n* = 33 (100)	(21 ± 2)	3 sessions: Stretch yoga (SYG) 1-hour; power yoga (PYG) 1-hour; and control (CG) 1-hour educational movie.	FS and FAS were administered at 3 time points for each session: pre, during, post-session.	Repeated Measures general Linear Model (RM GLM)	There was a significant main effect for condition time as well as a significant condition × time interaction effect. The univariate tests revealed that the condition effect was present for both the FS.	Strong
Vandoni et al. (2016)	Italy	Quasi-experimental	*n* = 27 (37)	Men (22.5 ± 2.0); women (23.3 ± 2.2)	2 group exercise training sessions at moderate and vigorous intensities.	FS and FAS immediately after the warm-up and immediately after the cooldown.	Repeated measures analysis of variance (ANOVA)	FS: a more positive affective response occurred during the moderate-intensity trial than during the vigorous-intensity trial. The changes in affective responses to exercise differed as a function of the exercise intensity trial. FAS: the changes in perceived activation during the exercise sessions were similar for the two exercise intensity trials. FAS increased (compared with 0-min values) at 15 min and 30 min, but not at 45 min and cooldown, for the two exercise intensity trials.	Strong

### Study Risk of Bias Assessment

The methodological quality of the selected studies was assessed using the Effective Public Health Practice Project (Thomas et al., 2004). This tool has empirical support for its utility in screening research articles (e.g., Armijo-Olivo et al., 2012), and it has been used in other physical activity contexts (e.g., Panão & Carraça, 2020; Thomas et al., 2020; Veldman et al., 2021). This quality assessment tool for quantitative studies is adjusted for experimental and non-experimental studies. This instrument comprises 22 questions assessing the quality of a study according to the following dimensions: selection bias, study design, confounders, blinding, data collection methods, withdrawals and drop-outs, intervention integrity, and statistical analyses. Two independent reviewers made this quality assessment. When reviewers disagreed, the results were compared, and the discrepancies were resolved by a third and external reviewer. All reviewers were debriefed and trained prior to the use of the quality checklist. Cohen’s inter-rater agreement presented an almost perfect agreement (k = .806; McHugh, 2012).

## Results

### Study Selection

**Figure 1.**
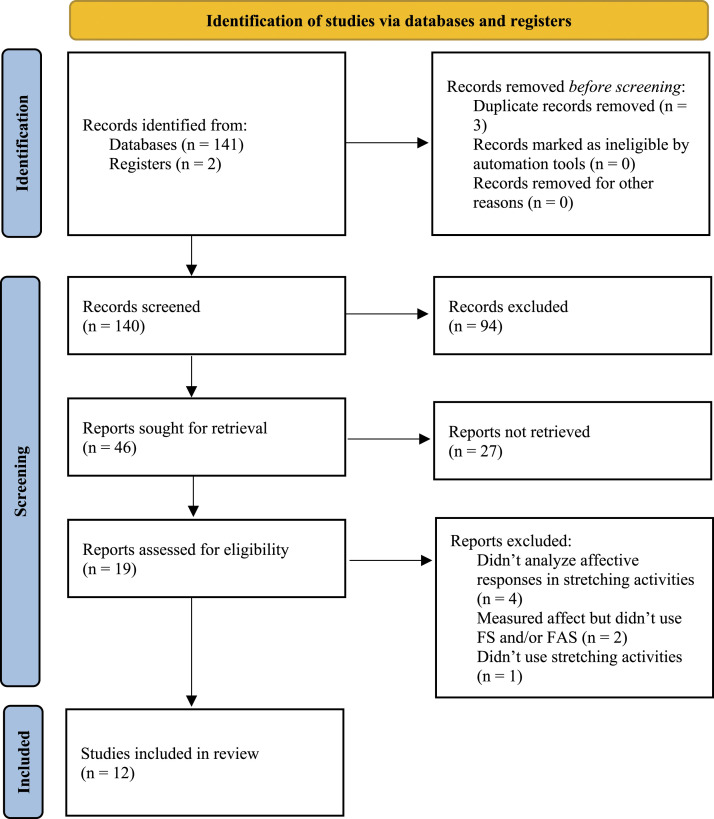
Flow Chart for Study Selection.

During the database search (see Figure 1), we identified a total of 141 titles. By analyzing bibliographical references from other sources, we added two additional potentially relevant studies. Three records were excluded as duplicates. We then screened these 140 records and excluded another 94 articles after reading their titles and realizing that the authors had different motives for their studies than those we intended. We carefully read the abstracts of the remaining 46 papers, and after this screening, we reduced the number by another 27 papers. We then read the full text of the 19 remaining papers and finally excluded seven of these that did not analyze affective responses in stretching activities. This left us with a final sample of 12 papers that we selected for thorough analysis.

### Study Summaries

The present review includes 12 empirical studies that evaluated participants’ affective responses in stretching-related activities that were published or accepted for publication up to February 2022. Table 1 represents a synthesis of the data extracted from these selected studies, listed alphabetically by the first author’s last name.

### Study Characteristics

Table 2 summarizes the descriptive data for the participant samples in the 12 papers analyzed. Most studies included convenience samples of active people, but some sampled sedentary or physically inactive individuals (Beltrão et al., 2020; McAuley, Jerome, Elavsky et al., 2003, McAuley, Jerome, Marquez et al., 2003). Edwards et al. (2018) did not provide information regarding participants characteristics. Participants met inclusion criteria and were engaged in a wide variety of physical activity contexts in which they used the Feeling Scale and/or Felt Arousal Scale in relation to stretching exercises. Altogether, there were 718 apparently healthy participants across the 12 studies. Overall, exercise was performed in distinct settings (recreational physical activity, laboratory setting) by varied participant age groups (young adults, adults, older adults) and involved different exercise modes (yoga, tai-chi, stretching, conditioning, dancing, individual stretching exercises, and group classes).

**Table 2.** Summary of Participant Characteristics.

Characteristics	Number of Studies	Population (*N* = 718)
Sample size		
<30	4	73
30–50	4	164
50–100	2	133
>100	2	348
Sex		
Female only	3	119
Male only	3	100
Mixed genders	6	499
Location		
North America	5	
South America	5	
Europe	1	
Asia	1	
Mean age (years)		
<25	7	239
25–64	2	61
≥65	2	418

### Risk of Bias in Studies

We assessed the studies’ methodological quality according to the Effective Public Health Practice Project (Thomas et al., 2004). Strong quality scores were due to better study design with fewer participant withdrawals and dropouts. Participant selection bias, confounding variables, and failures to anonymize aspects of the study from participants or evaluators were the bases for the weakest quality scores. Final tallies for the studies’ quality score category levels were as follows: (a) strong (4 studies), (b) moderate (3 studies), and week (5 studies). See Table 1.

### Categorical Descriptions of Study Characteristics

#### Exercise Mode

Among 12 studies in which participants engaged in stretching exercises, three studies involved individual stretching exercises (Barbosa et al., 2018; Beltrão et al., 2020; Melo et al., 2021), and seven involved stretching performed in group yoga classes (Follador et al., 2019; Reed, 2014; Sullivan et al., 2019), tai-chi (Chao et al., 2014; Follador et al., 2019), and guided stretching (Edwards et al., 2018; Follador et al., 2019; McAuley, Jerome, Elavsky et al., 2003; McAuley, Jerome, Marquez et al., 2003). Two studies included mainly aerobic exercise (Lee et al., 2021; Vandoni et al., 2016), in which we only analyzed those session moments when flexibility was assessed (i.e., pre-exercise, warm-up, and cooldown).

In the stretching classes/exercises, investigators used distinct approaches and types of contraction (i.e., static, dynamic, passive, active): Follador et al. (2019) used 16 static stretching exercises both in the upper and lower extremities; Melo et al. (2021) included static passive stretching for hamstring muscles; Beltrão et al. (2020) used static passive stretch exercise for the hamstrings; and Barbosa et al. (2018) divided groups according to static or dynamic hamstring stretches. In other studies, the type of stretch was not described (Edwards et al., 2018; Lee et al., 2021; McAuley, Jerome, Marquez et al., 2003; Vandoni et al., 2016).

#### Type of Study

Among all 12 studies included in this review, only one was not experimental (McAuley, Jerome, Elavsky et al., 2003). This study was an 18-month follow-up of McAuley, Jerome, Marquez et al. (2003). Five studies were randomized controlled trials (Barbosa et al., 2018; Beltrão et al., 2020; Edwards et al., 2018; McAuley, Jerome, Marquez et al., 2003; Melo et al., 2021), and six were quasi-experimental (Chao et al., 2014; Follador et al., 2019; Lee et al., 2021; Reed, 2014; Sullivan et al., 2019; Vandoni et al., 2016). In studies using a control/comparison group, groups were either divided by exercise mode (Barbosa et al., 2018; Edwards et al., 2018; Follador et al., 2019; McAuley, Jerome, Elavsky et al., 2003; McAuley, Jerome, Marquez et al., 2003) or exercise intensity (Beltrão et al., 2020; Melo et al., 2021). Reed (2014) compared two conditions (placebo vs. experimental) performing the same activity (introductory Hatha yoga).

#### FS/FAS Measurement Procedures

In only four studies did experimenters provide FS/FAS instructions and prior participant training for familiarity of the questionnaires (Beltrão et al., 2020; Chao et al., 2014; Follador et al., 2019; Vandoni et al., 2016). Measurement timings differed across studies, with five studies applying affective measurements only after the exercise session (Barbosa et al., 2018; Beltrão et al., 2020; McAuley, Jerome, Elavsky et al., 2003; McAuley, Jerome, Marquez et al., 2003; Melo et al., 2021), and all the remaining seven studies evaluating affective responses at baseline, during (the session, not during the stretching position), and post-session (Beltrão et al., 2020; Chao et al., 2014; Edwards et al., 2018; Follador et al., 2019; Lee et al., 2021; Reed, 2014; Sullivan et al., 2019; Vandoni et al., 2016). Across studies there was no apparent standardization of the time of assessment or number of measurements used in the protocol/session.

#### Intensity Control

Intensity of stretching varied from light to moderate, and seven studies used different instruments to control intensity. Three monitored intensity objectively through heart rate with a smartwatch (Edwards et al., 2018; Lee et al., 2021) or a heart monitor throughout the exercise session (Vandoni et al., 2016). Six studies measured intensity subjectively, with ratings of perceived exertion (Borg, 1982) used in four studies (Chao et al., 2014; Edwards et al., 2018; Follador et al., 2019; Vandoni et al., 2016), the scale of perceived effort in flexibility (PERFLEX; Dantas, 2008) used in one (Melo et al., 2021), and the Verbal Numerical Scale (Ferreira-Valente et al., 2011) also used in one (Beltrão et al., 2020). Barbosa et al. (2018) did not measure intensity, but asked participants to perform the stretching exercises until they felt mild discomfort. Sullivan et al. (2019) designed two yoga sessions differently, so that the power yoga session had a higher intensity and faster pace than the stretch yoga session. Three studies did not present intensity data (McAuley, Jerome, Elavsky et al., 2003; McAuley, Jerome, Marquez et al., 2003; Reed, 2014).

#### Transitional Affective States Plotted on the Circumplex Model

Follador et al. (2019), Lee et al. (2021), Sullivan et al. (2019), and Vandoni et al. (2016) assessed participants with both the FS and the FAS, and they plotted participants’ affective responses on the circumplex model. These four studies registered scores in two quadrants: low-activation pleasure (e.g., calmness, relaxation) and high-activation pleasure (e.g., energy, vigor). Follador et al. (2019) had three intervention groups perform tai-chi, yoga, or stretching, and they found that participants’ affective valence and arousal shifted from the low-activation pleasure quadrant (baseline) to the high-activation pleasure quadrant. Lee et al. (2021) asked participants to perform two low intensity and moderate intensity Zumba sessions, and they found that warm-up responses occurred in the low-activation pleasure quadrant for both intensities. Cooldown measures remained in the low-activation pleasure quadrant for the light intensity Zumba session and rose to the high-activation pleasure quadrant for the moderate intensity session. Sullivan et al. (2019) performed three interventions with the same participant group (power yoga, stretch yoga and control). In stretch yoga and control sessions, participants responses were only in the low-activation pleasure quadrant in all session stages (warm-up, fundamental stage, cooldown). In the power yoga session, participant responses were in the low-activation pleasure quadrant for baseline and post-session responses, but they were in the high-activation pleasure quadrant during the fundamental stage. In the study of Vandoni et al. (2016) the participants performed two group exercise sessions (moderate or vigorous intensity), but only the warm-up and cooldown measures were considered for this review. Warm-up affective responses were registered in the low-activation pleasure quadrant for both groups. While the moderate intensity group kept the same results in the cooldown measures as in the warm-up, the vigorous intensity group increased to the high-activation pleasure quadrant.

## Discussion

We sought in this review to assess whether affective responses measured with the FS and FAS in stretching activities in an ecologically natural physical activity context were useful and reflected any standardized methodological procedure. There was sufficient literature meeting our inclusion criteria and search and screening processes (*n* = 12 studies) to generate summary impressions and recommendations for future research. Among these 12 studies our quality assessment found four studies to be weak methodologically, three to be of moderate methodological quality, and five to have strong methodological quality. Participants had no problems comprehending the FS and FAS, despite their having undergone no familiarity training in most studies. As for the core affect assessment, there was no standardized timing (within a session or in relation to the stretch position) or frequency of assessment. Given this heterogeneity in assessment procedures in the protocols of these studies, several limitations emerge to our understanding of the overall trends or findings of prior research involving affect assessment in stretching activities. Additionally, an apparent disregard among these investigators of the conditions needed to effectively assess core affect (e.g., timing) highlights the need for future investigators to use standardized procedures that will allow accurate measurements, cross study comparisons, and a clear interpretation of the participants’ affective responses.

Timing has been shown to matter when assessing affect during aerobic and resistance exercise (Andrade et al., 2022; Cavarretta et al., 2019; Ekkekakis et al., 2011). According to the AHBF, the way a participant feels *during* the exercise is markedly different and often has an opposite valence to how they feel *after* the exercise. Indeed, while there is great variation in how individuals respond affectively during physical activity (Ladwig et al., 2017), post-physical activity affect is known to improve almost universally. This phenomenon is known as the “affective rebound effect” (Cavarretta et al., 2019; Ekkekakis et al., 2005, 2011), and it has been reported in resistance training (Cavarretta et al., 2019; Emanuel et al., 2021) and particularly in aerobic exercise, wherein a high percentage (>95%) of participants experience a positive affective response after the cessation of exercise (Ekkekakis et al., 2011). This should also be the case in stretching activities, due to the discomfort caused by the intrinsic characteristics of muscles under stretch. Yet, no study or methodological consideration of this issue was found in this review. This methodology gap in affect response measurement for stretching does not permit us to properly summarize results from studies reviewed in this article. For example, some of the included studies reported only post-exercise session measurements, while others, measured affect during the session/segment of the stretching activity (but none measured affect while the muscle was stretched); in other cases, assessments were made prior to and after the exercise session, excluding data regarding the affective panorama interpretation throughout the session. This poses a concern in interpreting data regarding the affective response to stretching exercise, since core affect can only be experienced in vivo, or in close proximity to the activity (Stevens et al., 2020; Zenko et al., 2016). Importantly, affect recall (i.e., affect assessment via memory of previous feelings subjected to cognitive appraisal) does not reflect core affect that emanated from exercise. Without this consideration, there may not be an accurate assessment of the in-session pleasure perception that, in past research, has been a relevant predictor of physical activity intention and later behavior (Rhodes & Kates, 2015). Given the absence of methodological standardization in core affect assessment and the apparent heterogeneity of these past approaches to studying stretching exercises, finding the best moment to assess the pleasure/displeasure responses in stretching activities should be a direct focus in future research.

Another relevant assessment variable is the frequency/number of assessments. To achieve an accurate assessment of the affective response, the FS and FAS should be used at regular intervals, balancing the accurate representation of affect during the session (or segments of the sessions; or some specific exercise modes) without burdening the participant with excessive assessments. When obtained in this way, this series of results can be plotted in the circumplex model of affect, thus allowing a deeper understanding of the affective response fluctuations across the session/activities. When this approach was used in four of the studies in this review, affective responses generally fell into both the low-activation pleasant quadrant and the high-activation pleasant quadrant, depending upon the intensities of the activity (e.g., light or moderate intensity), the distinct moments of the session (e.g., warm-up; cooldown), and the timings of the assessment in relation to the stretch (e.g., after, but not immediately after the stretch).

As seen here, the balance of assessments may vary with the exercise mode, duration, and intensity, among other variables. Still, the studies reviewed showed high heterogeneity in the number of affect assessments, which varied from one (Barbosa et al., 2018; McAuley, Jerome, Elavsky et al., 2003; McAuley, Jerome, Marquez et al., 2003; Melo et al., 2021) to seven (Lee et al., 2021) during the exercise sessions. Although the number of assessments can be set differently given each study’s aim and needs, the overall impression from this review is that discrepancies in the number of assessments performed creates challenges for making comparisons between studies and raises concerns regarding possible interpretations of affective response results. This problem may be increased by the fact that protocols used in these studies of stretching exercises involved a wide variety of exercise settings, including individual stretches versus group classes, and variance in warm-up versus and cooldown sections. Also, the investigators used diverse types of muscle contraction (i.e., static, dynamic, passive, active). Again, future researchers should attend to the development of methodological guidelines that allow an adjusted number of assessments to improve core affect evaluations.

On another relevant note, no problems were reported regarding the use and participants’ comprehension of the FS/FAS. This finding points to the feasibility/suitability of these scales for stretching activity and usual exercise contexts (e.g., health club activities), aligning this literature with what has been previously suggested in a related review in physical activity settings (Evmenenko & Teixeira, 2020). However, two issues must be considered regarding the scales’ feasibility in stretching activities. First, the fact that no measure has been taken during the stretch limits this interpretation, given that it may be easier to apply the scales while the exerciser is at rest (e.g., between stretches) versus during the stretch effort. Additionally, some stretching modes, like dynamic stretching, may not allow the scales’ application during the exercises, a consideration that must be contemplated when interpreting future efforts on the understanding of contextual feasibility. Second, the correct application and understanding of these scales is an important consideration. Duda (1998), Evmenenko and Teixeira (2020), and Zenko and Jones (2021) recommended that researchers and participants have prior training in scale administration to achieve consistent results. Among the studies reviewed here, four reported some previous participant training in the use of these scales (Beltrão et al., 2020; Chao et al., 2014; Lee et al., 2021; Vandoni et al., 2016), but no study reported experimenter training in this use. Given the specificity of these scales (and generally all perceptual scales; Haile et al., 2015), prior training in their use and interpretation should be standard procedure; this would help assure the quality of the assessments and the obtained data. This need may be particularly relevant in studies in which other scales are used in proximity (e.g., ratings of perceived exertion) to preclude participant confusion as, for example, in distinguishing between perceived effort and perceived activation/arousal. However, the needed extent of this training must be explored in future research, given that most studies included in our review did not mention the participants’ or applicants’ prior training in interpreting or applying these scales, and yet no negative reports on the use of the scales were made. This raises some concerns, as it is difficult to assess if this absence of information on the scales’ application and interpretation is due to effective or ineffective training in their use. Although at this point the feasibility/suitability of the scales seems adequate, additional studies are needed to substantiate this assumption.

### Limitations and Directions for Further Research

Although this review addressed a relevant and understudied aspect of affect assessment of stretching exercise/activity, future investigators should address methodological weaknesses we identified. First, there are a low number of studies that have specifically addressed affect responses to stretching activity, and there is high heterogeneity of experimental designs, research methods, and stretching activities and intensities, all of which limit the ability to summarize and interpret research results to date. In fact, it is still unclear how and when to assess core affect during stretching activities. For example, no study in this review explored the affective rebound when affect responses are measured *after* versus *during* stretching activities. While advancements in assessment procedures may take some time to be reflected in future research efforts, some attention can be given to the results of those studies who recorded the specific moment when the evaluation took place. However, because of these methodological problems in affective response assessment for stretching, we could not ascertain a summary perspective or interpretation of research to date in this field, and could only highlight priorities for future research.

## Conclusion

In summary, our findings indicated that core affect assessments with the FS and FAS in stretching-related activities still lack sufficient methodological support to generate summary impressions from research to date. Albeit with some reservations, we did find the administration of the FS and FAS to be feasible and useful in the most common contexts of research practice in this field, although no measurement was made during the stretch. Future investigators should design studies to discover and develop how, when, and how often affective responses assessment in stretching activities should occur. Most importantly, future research should address how positive affect associated with stretching activities affects future engagement or adherence in physical activity.

## References

[bibr1-00315125231160203] Ajzen I. (1991). The theory of planned behavior. Organizational Behavior and Human Decision Processes, 50(2), 179–211. 10.1016/0749-5978(91)90020-t

[bibr67-00315125231160203] Armijo-Olivo S. Stiles C. R. Hagen N. A. Biondo P. D. Cummings G. G. (2012). Assessment of study quality for systematic reviews: A comparison of the Cochrane Collaboration Risk of Bias Tool and the Effective Public Health Practice Project Quality Assessment Tool: methodological research. Journal of Evaluation in Clinical Practice, 18(1), 12–18. 10.1111/j.1365-2753.2010.01516.x 20698919

[bibr2-00315125231160203] Andrade A. J. Ekkekakis P. Evmenenko A. Monteiro D. Rodrigues F. Cid L. Teixeira D. S. (2022). Affective responses to resistance exercise: Toward a consensus on the timing of assessments. Psychology of Sport and Exercise, 62, 102223. 10.1016/j.psychsport.2022.102223 37665925

[bibr3-00315125231160203] Bandura A. (1998). Health promotion from the perspective of social cognitive theory. Psychology & Health, 13(4), 623–649. 10.1080/08870449808407422

[bibr4-00315125231160203] Barbosa G. M. Figueirêdo Dantas G. A. Silva B. R. Souza T. O. Brito Vieira W. H. (2018). Static or dynamic stretching program does not change the acute responses of neuromuscular and functional performance in healthy subjects: A single-blind randomized controlled trial. Revista Brasileira de Ciências do Esporte, 40(4), 418–426. 10.1016/j.rbce.2018.06.002

[bibr5-00315125231160203] Beltrão N. B. Ximenes Santos C. de Oliveira V. M. A. Pirauá A. L. T. Behm D. Pitangui A. C. R. de Araújo R. C. (2020). Effects of a 12-week chronic stretch training program at different intensities on joint and muscle mechanical responses: A randomized clinical trial. Journal of Sport Rehabilitation, 29(7), 904–912. 10.1123/jsr.2018-0443 31648203

[bibr64-00315125231160203] Booth A. Clarke M. Dooley G. Ghersi D. Moher D. Petticrew M. Stewart L. (2012). The nuts and bolts of PROSPERO: An international prospective register of systematic reviews. Systematic reviews, 1, 2. 10.1186/2046-4053-1-2 22587842 PMC3348673

[bibr6-00315125231160203] Borg G. A. (1982). Psychophysical bases of perceived exertion. Medicine & Science in Sports & Exercise, 14(5), 377–381. 10.1249/00005768-198205000-00012 7154893

[bibr7-00315125231160203] Brand R. Ekkekakis P. (2018). Affective–reflective theory of physical inactivity and exercise. German Journal of Exercise and Sport Research, 48(1), 48–58. 10.1007/s12662-017-0477-9

[bibr8-00315125231160203] Cavarretta D. J. Hall E. E. Bixby W. R. (2019). Affective responses from different modalities of resistance exercise: Timing matters. Frontiers in Sports and Active Living, 1, 5. 10.3389/fspor.2019.00005 33344929 PMC7739567

[bibr9-00315125231160203] Chao C. H. N. Costa E. C. Okano A. H. De Brito Farias T. Farias L. F. Elsangedy H. M. Krinski K. (2014). Rating of perceived exertion and affective responses during Tai Chi Chuan. Perceptual and Motor Skills, 118(3), 926–939. 10.2466/10.06.PMS.118k27w5 25068755

[bibr10-00315125231160203] Cheval B. Boisgontier M. P. (2021). The theory of effort minimization in physical activity. Exercise and Sport Sciences Reviews, 49(3), 168–178. 10.1249/JES.0000000000000252 34112744 PMC8191473

[bibr11-00315125231160203] Cruz-Ferreira A. Fernandes J. Laranjo L. Bernardo L. M. Silva A. (2011). A systematic review of the effects of pilates method of exercise in healthy people. Archives of Physical Medicine and Rehabilitation, 92(12), 2071–2081. 10.1016/j.apmr.2011.06.018 22030232

[bibr12-00315125231160203] DiGiacomo M. Lam P. Roberts B. L. Lau T. C. Song R. Davidson P. M. (2010). Exploring the reasons for adherence to t'ai chi practice. Journal of Alternative and Complementary Medicine, 16(12), 1245–1246. 10.1089/acm.2010.0510 21114405

[bibr13-00315125231160203] Duda J. (1998). Advances in sport and exercise psychology measurement. Fitness Information Technology.

[bibr14-00315125231160203] Edwards M. K. Rhodes R. E. Mann J. R. Loprinzi P. D. (2018). Effects of acute aerobic exercise or meditation on emotional regulation. Physiology & Behavior, 186, 16–24. 10.1016/j.physbeh.2017.12.037 29309746

[bibr15-00315125231160203] Ekkekakis P. Hall E. E. Petruzzello S. J. (2005). Variation and homogeneity in affective responses to physical activity of varying intensities: An alternative perspective on dose-response based on evolutionary considerations. Journal of Sports Sciences, 23(5), 477–500. 10.1080/02640410400021492 16194996

[bibr16-00315125231160203] Ekkekakis P. Parfitt G. Petruzzello S. J. (2011). The pleasure and displeasure people feel when they exercise at different intensities: Decennial update and progress towards a tripartite rationale for exercise intensity prescription. Sports Medicine, 41(8), 641–671. 10.2165/11590680-000000000-00000 21780850

[bibr17-00315125231160203] Ekkekakis P. Petruzzello S. J. (2000). Analysis of the affect measurement conundrum in exercise psychology: I. Fundamental issues. Psychology of Sport and Exercise, 1(2), 71–88. 10.1016/s1469-0292(00)00010-8

[bibr18-00315125231160203] Ekkekakis P. Zenko Z. (2016). Chapter 18 - Escape from cognitivism: Exercise as hedonic experience. In Raab M. Wylleman P. Seiler R. Elbe A.-M. Hatzigeorgiadis A. (Eds.), Sport and exercise psychology research (pp. 389–414). Academic Press. 10.1016/B978-0-12-803634-1.00018-2

[bibr19-00315125231160203] Emanuel A. Rozen Smukas I. Halperin I. (2021). How one feels during resistance exercises: A repetition-by-repetition analysis across exercises and loads. International Journal of Sports Physiology and Performance, 16(1), 135–144. 10.1123/ijspp.2019-0733 32781441

[bibr20-00315125231160203] Evmenenko A. Teixeira D. S. (2020). The circumplex model of affect in physical activity contexts: A systematic review. International Journal of Sport and Exercise Psychology, 20(1), 168–201. 10.1080/1612197X.2020.1854818

[bibr21-00315125231160203] Fernández-Rodríguez R. Álvarez-Bueno C. Ferri-Morales A. Torres-Costoso A. I. Cavero-Redondo I. Martínez-Vizcaíno V. (2019). Pilates method improves cardiorespiratory fitness: A systematic review and meta-analysis. Journal of Clinical Medicine, 8(11), 1761. 10.3390/jcm8111761 31652806 PMC6912807

[bibr22-00315125231160203] Ferreira-Valente M. A. Pais-Ribeiro J. L. Jensen M. P. (2011). Validity of four pain intensity rating scales. Pain, 152(10), 2399–2404. 10.1016/j.pain.2011.07.005 21856077

[bibr23-00315125231160203] Follador L. Alves R. C. Ferreira S. D. S. Silva A. C. Silva S. G. D. (2019). Perceived exertion and affect from tai chi, yoga, and stretching classes for Elderly women. Perceptual and Motor Skills, 126(2), 223–240. 10.1177/0031512518823661 30638426

[bibr24-00315125231160203] Guthold R. Stevens G. A. Riley L. M. Bull F. C. (2018). Worldwide trends in insufficient physical activity from 2001 to 2016: A pooled analysis of 358 population-based surveys with 1·9 million participants. The Lancet. Global Health, 6(10), Article e1077-e1086. 10.1016/S2214-109X(18)30357-7 30193830

[bibr25-00315125231160203] Haile L. Gallagher M. Jr. Robertson R. J. (2015). Perceived exertion laboratory manual: From standard practice to contemporary application. Springer Science + Business Media. 10.1007/978-1-4939-1917-8

[bibr26-00315125231160203] Hardy C. J. Rejeski W. J. (1989). Not what, but how one feels: The measurement of affect during exercise. Journal of Sport and Exercise Psychology, 11(3), 304–317. 10.1123/jsep.11.3.304

[bibr65-00315125231160203] Higgins J. P. T. Green S. (2013). Cochrane Handbook for Systematic Reviews of Interventions, Version 5.1.0. The Cochrane Collaboration.

[bibr27-00315125231160203] Ladwig M. A. Hartman M. E. Ekkekakis P. (2017). Affect-based exercise prescription: An idea whose time has come? ACSM’s Health & Fitness Journal, 21(5), 10–15. 10.1249/fit.0000000000000332

[bibr28-00315125231160203] Lee J. Park J. Kim Y. Woo M. (2021). Affective change with variations in Zumba fitness intensity as measured by a smartwatch. Perceptual and Motor Skills, 128(5), 2255–2278. 10.1177/00315125211022700 34120521

[bibr29-00315125231160203] Martin Dantas E. H. (2008). Scale of perceived exertion in the flexibility (PERFLEX): A dimensionless tool to evaluate the intensity? Fitness & Performance Journal, 7(5), 289–294. 10.3900/fpj.7.5.289.e

[bibr30-00315125231160203] McAuley E. Jerome G. J. Elavsky S. Marquez D. X. Ramsey S. N. (2003a). Predicting long-term maintenance of physical activity in older adults. Preventive Medicine, 37(2), 110–118. 10.1016/s0091-7435(03)00089-6 12855210

[bibr31-00315125231160203] McAuley E. Jerome G. J. Marquez D. X. Elavsky S. Blissmer B. (2003b). Exercise self-efficacy in older adults: Social, affective, and behavioral influences. Annals of Behavioral Medicine: A Publication of the Society of Behavioral Medicine, 25(1), 1–7. 10.1207/S15324796ABM2501_01 12581930

[bibr32-00315125231160203] McHugh M. L. (2012). Interrater reliability: The kappa statistic. Biochemia Medica, 22(3), 276–282. 10.11613/bm.2012.031 23092060 PMC3900052

[bibr33-00315125231160203] Melo R. R. V. Cerqueira M. S. Barbosa G. M. Laurentino A. L. B. A. Franca I. M. Souza T. O. Brito Vieira W. H. (2021). Static stretching at pain-tolerated intensity is not necessary to increase knee range of motion in amateur soccer players: A randomized trial. Muscle Ligaments and Tendons Journal, 11(3), 536–546. 10.32098/mltj.03.2021.19

[bibr66-00315125231160203] Methley A. M. Campbell S. Chew-Graham C. McNally R. Cheraghi-Sohi S. (2014). PICO, PICOS, and SPIDER: A comparison study of specificity and sensitivity in three search tools for qualitative systematic reviews. BMC Health Services Research, 14, 579. 10.1186/s12913-014-0579-0 25413154 PMC4310146

[bibr34-00315125231160203] Miyamoto G. C. Costa L. O. P. Cabral C. M. N. (2013). Efficacy of the pilates method for pain and disability in patients with chronic nonspecific low back pain: A systematic review with meta-analysis. Brazilian Journal of Physical Therapy, 17(6), 517–532. 10.1590/S1413-35552012005000127 24346291 PMC4207151

[bibr35-00315125231160203] Nikitara K. Odani S. Demenagas N. Rachiotis G. Symvoulakis E. Vardavas C. (2021). Prevalence and correlates of physical inactivity in adults across 28 European countries. European Journal of Public Health, 31(4), 840–845. 10.1093/eurpub/ckab067 34000007 PMC8504996

[bibr36-00315125231160203] Opplert J. Babault N. (2018). Acute effects of dynamic stretching on muscle flexibility and performance: An analysis of the current literature. Sports Medicine, 48(2), 299–325. 10.1007/s40279-017-0797-9 29063454

[bibr37-00315125231160203] Page M. J. Moher D. Bossuyt P. M. Boutron I. Hoffmann T. C. Mulrow C. D. Shamseer L. Tetzlaff J. M. Akl E. A. Brennan S. E. Chou R. Glanville J. Grimshaw J. M. Hrobjartsson A. Lalu M. M. Li T. Loder E. W. Mayo-Wilson E. McDonald S. McKenzie J. E. (2021). PRISMA 2020 explanation and elaboration: Updated guidance and exemplars for reporting systematic reviews. BMJ, 372, n160. 10.1136/bmj.n160 33781993 PMC8005925

[bibr68-00315125231160203] Panão I. Carraça E. V. (2020). Effects of exercise motivations on body image and eating habits/behaviours: A systematic review. Nutrition & Dietetics: The Journal of the Dietitians Association of Australia, 77(1), 41–59. 10.1111/1747-0080.12575 31449357

[bibr38-00315125231160203] Prochaska J. O. Velicer W. F. (1997). The transtheoretical model of health behavior change. American Journal of Health Promotion: AJHP, 12(1), 38–48. 10.4278/0890-1171-12.1.38 10170434

[bibr39-00315125231160203] Rebar A. L. Dimmock J. A. Jackson B. Rhodes R. E. Kates A. Starling J. Vandelanotte C. (2016). A systematic review of the effects of non-conscious regulatory processes in physical activity. Health Psychology Review, 10(4), 395–407. 10.1080/17437199.2016.1183505 27118430

[bibr40-00315125231160203] Reed J. (2014). Effect of placebo-induced changes in Expectancies on self-reported affect associated with yoga practice. Journal of Sport Behavior, 37(3), 268–285.

[bibr41-00315125231160203] Rhodes R. E. Kates A. (2015). Can the affective response to exercise predict future motives and physical activity behavior? A systematic review of published evidence. Annals of Behavioral Medicine: A Publication of the Society of Behavioral Medicine, 49(5), 715–731. 10.1007/s12160-015-9704-5 25921307

[bibr42-00315125231160203] Riebe D. Ehrman J. Liguori G. Magal M. (2018). ACSM’s guidelines for exercise testing and prescription (10th ed.). In Kluwer W. (Ed.). American College of Sports Medicine.

[bibr43-00315125231160203] Rodrigues F. Teixeira D. S. Neiva H. P. Cid L. Monteiro D. (2020). Understanding exercise adherence: The predictability of past experience and motivational determinants. Brain Sciences, 10(2), 98. 10.3390/brainsci10020098 32059352 PMC7071831

[bibr44-00315125231160203] Russell J. A. (1980). A circumplex model of affect. Journal of Personality and Social Psychology, 39(6), 1161–1178. 10.1037/h0077714

[bibr45-00315125231160203] Smith C. Hancock H. Blake-Mortimer J. Eckert K. (2007). A randomised comparative trial of yoga and relaxation to reduce stress and anxiety. Complementary Therapies in Medicine, 15(2), 77–83. 10.1016/j.ctim.2006.05.001 17544857

[bibr46-00315125231160203] Stevens C. J. Baldwin A. S. Bryan A. D. Conner M. Rhodes R. E. Williams D. M. (2020). Affective determinants of physical activity: A conceptual framework and narrative review. Frontiers in Psychology, 11, 568331. 10.3389/fpsyg.2020.568331 33335497 PMC7735992

[bibr47-00315125231160203] Strobach T. Englert C. Jekauc D. Pfeffer I. (2020). Predicting adoption and maintenance of physical activity in the context of dual-process theories. Performance Enhancement & Health, 8(1), 100162. 10.1016/j.peh.2020.100162

[bibr48-00315125231160203] Sullivan M. Carberry A. Evans E. S. Hall E. E. Nepocatych S. (2019). The effects of power and stretch yoga on affect and salivary cortisol in women. Journal of Health Psychology, 24(12), 1658–1667. 10.1177/1359105317694487 28810420

[bibr49-00315125231160203] Svebak S. Murgatroyd S. (1985). Metamotivational dominance. A multimethod validation of reversal theory constructs. Journal of Personality and Social Psychology, 48(1), 107–116. 10.1037/0022-3514.48.1.107

[bibr50-00315125231160203] Telles S. Singh N. Yadav A. Balkrishna A. (2012). Effect of yoga on different aspects of mental health. Indian Journal of Physiology and Pharmacology, 56(3), 245–254. 23734439

[bibr51-00315125231160203] Thomas B. H. Ciliska D. Dobbins M. Micucci S. (2004). A process for systematically reviewing the literature: Providing the research evidence for public health nursing interventions. Worldviews on Evidence-Based Nursing, 1(3), 176–184. 10.1111/j.1524-475X.2004.04006.x 17163895

[bibr70-00315125231160203] Thomas J. Thirlaway K. Bowes N. Meyers R. (2020). Effects of combining physical activity with psychotherapy on mental health and well-being: A systematic review. Journal of Affective Disorders, 265, 475–485. 10.1016/j.jad.2020.01.070 32090775

[bibr52-00315125231160203] Thompson W. R. (2022). Worldwide survey of fitness trends for 2022. ACSM’s Health & Fitness Journal, 26(1), 11–20. 10.1249/fit.0000000000000732

[bibr53-00315125231160203] Vandoni M. Codrons E. Marin L. Correale L. Bigliassi M. Buzzachera C. F. (2016). Psychophysiological responses to group exercise training sessions: Does exercise intensity matter? PLoS One, 11(8), Article e0149997. 10.1371/journal.pone.0149997 27490493 PMC4973874

[bibr69-00315125231160203] Veldman S. L. C. Chin A Paw M. G. M. Altenburg T. M. (2021). Physical activity and prospective associations with indicators of health and development in children aged <5 years: A systematic review. The International Journal of Behavioral Nutrition and Physical Activity, 18(1), 6. 10.1186/s12966-020-01072-w 33413484 PMC7791660

[bibr54-00315125231160203] Wang C. Bannuru R. Ramel J. Kupelnick B. Scott T. Schmid C. H. (2010). Tai Chi on psychological well-being: Systematic review and meta-analysis. BMC Complementary and Alternative Medicine, 10(1), 23. 10.1186/1472-6882-10-23 20492638 PMC2893078

[bibr55-00315125231160203] Warburton D. E. R. Bredin S. S. D. (2017). Health benefits of physical activity: A systematic review of current systematic reviews. Current Opinion in Cardiology, 32(5), 541–556. 10.1097/HCO.0000000000000437 28708630

[bibr56-00315125231160203] Warburton D. E. R. Nicol C. W. Bredin S. S. D. (2006). Health benefits of physical activity: The evidence. CMAJ, 174(6), 801–809. 10.1503/cmaj.051351 16534088 PMC1402378

[bibr57-00315125231160203] Williams D. M. (2008). Exercise, affect, and adherence: An integrated model and a case for self-paced exercise. Journal of Sport & Exercise Psychology, 30(5), 471–496. 10.1123/jsep.30.5.471 18971508 PMC4222174

[bibr58-00315125231160203] Williams D. M. Evans D. R. (2014). Current emotion research in health behavior science. Emotion Review, 6(3), 277–287. 10.1177/1754073914523052 PMC12975116 41815188

[bibr59-00315125231160203] Yeh G. Y. Wang C. Wayne P. M. Phillips R. (2009). Tai Chi exercise for patients with cardiovascular conditions and risk factors: A systematic review. Journal of Cardiopulmonary Rehabilitation and Prevention, 29(3), 152–160. 10.1097/HCR.0b013e3181a33379 19471133 PMC2755083

[bibr60-00315125231160203] Yeh G. Y. Wang C. Wayne P. M. Phillips R. S. (2008). The effect of tai chi exercise on blood pressure: A systematic review. Preventive Cardiology, 11(2), 82–89. 10.1111/j.1751-7141.2008.07565.x 18401235

[bibr61-00315125231160203] Yoshihara K. Hiramoto T. Oka T. Kubo C. Sudo N. (2014). Effect of 12 weeks of yoga training on the somatization, psychological symptoms, and stress-related biomarkers of healthy women. BioPsychoSocial Medicine, 8(1), 1. 10.1186/1751-0759-8-1 24383884 PMC3892034

[bibr62-00315125231160203] Zenko Z. Ekkekakis P. Ariely D. (2016). Can you have your vigorous exercise and Enjoy it too? Ramping intensity down increases postexercise, remembered, and forecasted pleasure. Journal of Sport & Exercise Psychology, 38(2), 149–159. 10.1123/jsep.2015-0286 27390185

[bibr63-00315125231160203] Zenko Z. Jones L. (Eds), (2021). Essentials of exercise and sport psychology: An open access textbook. Society for Transparency, Openness, and Replication in Kinesiology. 10.51224/B1000

